# Fatigue in Metabolic Dysfunction-Associated Steatotic Liver Disease: Links to Muscle Function, Hypoxia, and Hypertension †

**DOI:** 10.3390/healthcare13172206

**Published:** 2025-09-03

**Authors:** Anna F. Sheptulina, Adel A. Yafarova, Elvira M. Mamutova, Oxana M. Drapkina

**Affiliations:** 1 Laboratory of Experimental and Preventive Gastroenterology, National Medical Research Center for Therapy and Preventive Medicine, Moscow 101990, Russia; adeleyafaroff@gmail.com (A.A.Y.); emamutova@gnicpm.ru (E.M.M.); 2 Department of Fundamental and Applied Aspects of Obesity, National Medical Research Center for Therapy and Preventive Medicine, Moscow 101990, Russia; drapkina@bk.ru

**Keywords:** metabolic dysfunction-associated steatotic liver disease (MASLD), fatigue, hypoxia, muscle strength, blood pressure (BP)

## Abstract

Background/Objectives: Fatigue is the most common systemic manifestation of chronic liver diseases, including metabolic dysfunction-associated steatotic liver disease (MASLD). Fatigue not only adversely affects quality of life in MASLD patients but also complicates the attainment of therapeutic goals and contributes to a worse prognosis. This study aimed to analyze the relationship between clinically significant fatigue and laboratory parameters reflecting systemic inflammation, liver function, body composition, muscle strength, and blood pressure in patients with MASLD. Methods: A total of 154 patients with a confirmed diagnosis of MASLD were enrolled in this study. All participants underwent anthropometric assessment, laboratory testing, abdominal ultrasonography, and point shear-wave elastography. Muscle strength was evaluated using handgrip strength (GS) measurement and the Five Times Sit-to-Stand Test (5TSTS). Skeletal muscle mass (SMM) was quantified using dual-energy X-ray absorptiometry (DXA). Fatigue was evaluated using the Fatigue Assessment Scale (FAS), with scores ≥ 22 indicating clinically significant fatigue. Results: Patients with FAS scores ≥ 22 exhibited significantly lower hemoglobin levels (*p* = 0.004) and erythrocyte counts (*p* = 0.011), along with a significantly elevated erythrocyte sedimentation rate (ESR; *p* = 0.002) and C-reactive protein level (CRP; *p* = 0.007). Furthermore, MASLD patients with FAS scores ≥ 22 demonstrated significantly reduced relative grip strength (*p* = 0.012) and took longer to complete the 5TSTS (*p* = 0.011). Additionally, these patients had higher maximum systolic and diastolic blood pressure values compared to those with FAS scores < 22 (*p* = 0.028 and *p* = 0.019, respectively). Conclusions: These findings underscore the multifactorial nature of fatigue in MASLD and highlight the need for a comprehensive management strategy. Such a strategy should include dietary modification, increased physical activity, targeted treatment of systemic manifestations of MASLD, and appropriate management of comorbidities.

## 1. Introduction

Metabolic dysfunction-associated steatotic liver disease (MASLD) affects nearly one-third of the global population, with an estimated prevalence of 32.4% [1]. Over the past decade, the prevalence of MASLD has increased substantially, rising from 25.5% in 2005 to 37.8% in 2016, with an estimated annual incidence of 46.9 new cases per 1000 person-years [1,2]. Beyond the risk of progression to steatohepatitis, fibrosis, and cirrhosis, MASLD is increasingly recognized as a systemic condition associated with a wide range of extrahepatic manifestations. Among them, clinically significant fatigue has emerged as one of the most common and debilitating, affecting 51.1–67.8% of patients [3,4].

Fatigue in MASLD is a complex, multifactorial symptom that substantially impairs physical functioning, social participation, emotional well-being, and work performance. Evidence from the multinational LISTEN-MASLD study, which analyzed 1600 patient narratives across eight countries, identified fatigue as the single most frequently reported symptom, accounting for 20% of all complaints [5]. Notably, fatigue in MASLD is not consistently related to histological disease severity or insulin resistance [6], suggesting that its pathogenesis extends beyond liver involvement alone.

Several interrelated mechanisms contribute to fatigue in MASLD. Autonomic nervous system dysfunction is common in these patients, manifesting as orthostatic hypotension, vasovagal responses, and impaired nocturnal blood pressure regulation, which together reduce adaptive capacity and aggravate fatigue [7]. Importantly, autonomic dysfunction is also a hallmark of hypertension (HTN)—a frequent comorbidity of MASLD—providing a pathophysiological link between the two conditions [8,9]. Inflammation further contributes to fatigue, with elevated levels of proinflammatory cytokines such as interleukin (IL)-1, IL-6, IL-18, and tumor necrosis factor-alpha (TNF-α) being closely associated with symptom severity [9].

Another key factor is sarcopenia, which is often present in MASLD patients, particularly those with obesity. Sarcopenia, characterized by reduced muscle strength and endurance, decreases exercise tolerance and contributes to fatigue via psychological mechanisms. Indeed, muscle loss and dynapenia have been shown to correlate not only with fatigue but also with depressive symptoms, independent of age and activity levels [10]. The coexistence of sarcopenia, MASLD, and HTN creates an especially unfavorable metabolic phenotype characterized by impaired hemodynamics, decreased muscle perfusion, accelerated neuromuscular fatigue, and elevated inflammatory burden [11,12,13,14]. In addition, psychosocial factors such as disease-related and weight-related stigma may amplify fatigue by contributing to stress, depression, and social withdrawal [15].

Taken together, these findings highlight fatigue as a key but underrecognized manifestation of MASLD with a multifactorial genesis encompassing metabolic, vascular, inflammatory, musculoskeletal, and psychological domains. Given its high prevalence and substantial impact on quality of life, early recognition of fatigue in MASLD is essential. Proactive identification of fatigue can inform the design of targeted lifestyle interventions—including personalized exercise programs and nutritional strategies—aimed at mitigating its impact and improving patient outcomes. The present study therefore sought to investigate the relationship between clinically significant fatigue and laboratory markers of systemic inflammation, liver function, body composition, and muscle strength in patients with MASLD.

## 2. Materials and Methods

### 2.1. Subjects

This cross-sectional study was carried out at the National Medical Research Center for Therapy and Preventive Medicine, Ministry of Health of the Russian Federation. Consecutive patients with a confirmed diagnosis of MASLD who met all inclusion criteria and none of the exclusion criteria were enrolled. The enrollment period spanned February 2024 to January 2025. The study protocol was approved by the local ethics committee (Protocol No. 08-03/22, dated 27 December 2022). Written informed consent was obtained from all participants prior to enrollment.

Eligible participants met the following criteria:

(1) age between 20 and 70 years;
(2) ultrasound findings consistent with MASLD (detailed in Section 2.2);
(3) presence of at least one cardiometabolic risk factor, as outlined in reference [16].

Exclusion criteria included alcohol abuse (RUS-AUDIT score ≥ 8; RUS-AUDIT is the Russian-language version of the Alcohol Use Disorders Identification Test), liver diseases of other etiologies (based on medical history and documentation), current or recent (within 4 weeks) use of anti-obesity medications, acute infectious diseases, decompensated chronic cardiovascular, renal, or hepatic conditions, active malignancies without curative treatment, ongoing or recently completed (within 4 weeks prior to enrollment) antineoplastic therapy, connective tissue diseases, inflammatory bowel disease, type 1 diabetes mellitus, a history of lower limb fractures within the 6 months preceding this study, if accompanied by persistent functional impairment, and any clinically significant conditions impairing mobility or self-care. Pregnancy and breastfeeding were also exclusion criteria.

All study procedures—including medical history collection, administration of the Fatigue Assessment Scale (FAS), physical examination, anthropometric measurements (weight, height, waist circumference [WC], body mass index [BMI]), laboratory tests (complete blood count and biochemical analyses), abdominal ultrasound, muscle strength assessment, and dual-energy X-ray absorptiometry (DXA)—were carried out during a single visit or over two visits to the center, with an interval not exceeding five days.

### 2.2. Diagnostic Approach to Metabolic Dysfunction-Associated Steatotic Liver Disease

The diagnosis of MASLD was established according to the 2023 international consensus criteria [16]. Hepatic steatosis was identified on abdominal ultrasound, defined by at least one of the following features: (1) increased echogenicity of the liver parenchyma compared with the renal cortex; (2) attenuation of the vascular pattern; or (3) posterior beam attenuation with reduced visualization of the liver capsule and diaphragm. Ultrasound examinations were performed following a standardized protocol using a Philips Affiniti 70 system (Philips, Amsterdam, The Netherlands).

Presence of at least one cardiometabolic risk factor, as outlined in [17], served as the second diagnostic criterion.

All patients were screened for hepatitis B and C viral markers (HBsAg and anti-HCV antibodies) and assessed for alcohol consumption patterns using the RUS-AUDIT questionnaire, the Russian-language version of the Alcohol Use Disorders Identification Test (AUDIT) [2].

To further assess the probability of hepatic steatosis, the Fatty Liver Index (FLI) was calculated. Steatosis was excluded at FLI < 30 and diagnosed at FLI ≥ 60 [17].

Liver stiffness was assessed using point shear-wave elastography (pSWE) with a convex C5-1 transducer on the Philips Affiniti 70 system (Philips, Amsterdam, The Netherlands). Examinations were conducted according to established reliability criteria for liver stiffness measurement. Liver stiffness values below 7 kPa were interpreted as indicating the absence of significant fibrosis (F ≤ 3 on the METAVIR scale); values of 12 kPa or greater were consistent with liver cirrhosis (F4); and values between 7 and 12 kPa—the ‘gray zone’—necessitated re-evaluation after one to two months [18,19].

### 2.3. Anthropometric Parameters

Body weight was assessed using calibrated electronic floor scales (VMEN-200-50/100-I-ST-A; TVES LLC, Tambov Oblast, Russia) placed on a flat, stable surface. Standing height was measured with a stadiometer (R-St-MSK, MSK-234; Medstalkonstruktsiya LLC, Ufa, Russia) according to standard procedures. BMI was calculated as weight in kilograms divided by height in meters squared (kg/m^2^).

WC was measured with participants standing upright, abdomen relaxed, arms hanging naturally at the sides, and heels together. The measurement was taken at the narrowest point of the torso (natural waist) at the end of a normal expiration, using a flexible tape placed close to the clothing but without compressing the skin.

### 2.4. Body Composition Assessment

Body composition was evaluated with the Lunar iDXA system (General Electric HealthCare, Chicago, IL, USA) using the enCORE software, version 18.0. The following parameters were obtained: skeletal muscle mass (SMM); appendicular skeletal muscle mass (ALM), defined as the combined SMM of both arms and legs; total fat mass; visceral and subcutaneous adipose tissue mass; and body fat percentage. The appendicular lean mass-to-body-weight ratio (ALM/W) was calculated as the sum of lean mass in the upper and lower extremities, normalized to body weight. Low SMM was defined as an ALM/W < 28.27% in men and <23.47% in women [20].

### 2.5. Measurement of Muscle Strength

Upper- and lower-limb strength were assessed using handgrip strength (GS) and the Five Times Sit-to-Stand Test (5TSTS). GS of the dominant hand was measured with a mechanical dynamometer (DK-50; NTMIZ CJSC, Nizhny Tagil, Russia). Participants performed three maximal trials with 60 s rest intervals; the highest value was used. Low GS was defined as ≤30 kg (men, BMI 26.1–28 kg/m^2^), ≤32 kg (men, BMI > 28 kg/m^2^), ≤18 kg (women, BMI 26.1–29 kg/m^2^), and ≤21 kg (women, BMI > 29 kg/m^2^) [21]. Relative GS (rGS) was calculated as the ratio of maximal GS (kg) to BMI (kg/m^2^) [22].

All patients also completed the 5TSTS according to the previously described methodology [23,24]. A prolonged completion time (>15 s) was indicative of reduced muscle strength [23].

### 2.6. Assessment of Fatigue

The FAS was used for the evaluation of fatigue. The FAS independently evaluates both mental and physical components of fatigue, regardless of neuroticism or depression. The FAS has demonstrated high validity for detecting fatigue in clinical research settings [25]. According to K. Bhandari et al. [26], the FAS is recommended for assessing fatigue severity in patients with chronic liver disease. The scale comprises 10 items with multiple-choice responses, where participants select the option that best reflects their condition over the past month. Total scores range from 10 to 50 points, with scores of ≥22 indicating clinically significant fatigue [27].

### 2.7. Statistical Methods

The Kolmogorov–Smirnov test was applied to assess data distribution, which was non-normal; therefore, nonparametric methods were used. Continuous variables are reported as medians with interquartile ranges (IQR, 25th–75th percentiles), and categorical variables as counts and percentages (*n*, %). Between-group comparisons were performed using the Mann–Whitney U test, and associations between continuous variables were assessed with Spearman’s rank correlation coefficient (ρ). Correlation strength was classified according to the Chaddock scale: negligible (0.1 ≤ ρ < 0.3), weak (0.3 ≤ ρ < 0.5), moderate (0.5 ≤ ρ < 0.7), strong (0.7 ≤ ρ < 0.9), and very strong (0.9 ≤ ρ < 1.0) [28]. Categorical variables were compared using Pearson’s *χ*^2^ test. Logistic regression analysis was performed to determine independent predictors of clinically significant fatigue. A two-sided *p*-value < 0.05 was considered statistically significant. Analyses were conducted using IBM SPSS Statistics, version 27.0 (IBM Corp., Armonk, NY, USA).

## 3. Results

### 3.1. General Characteristics of Patients

**Figure 1:**
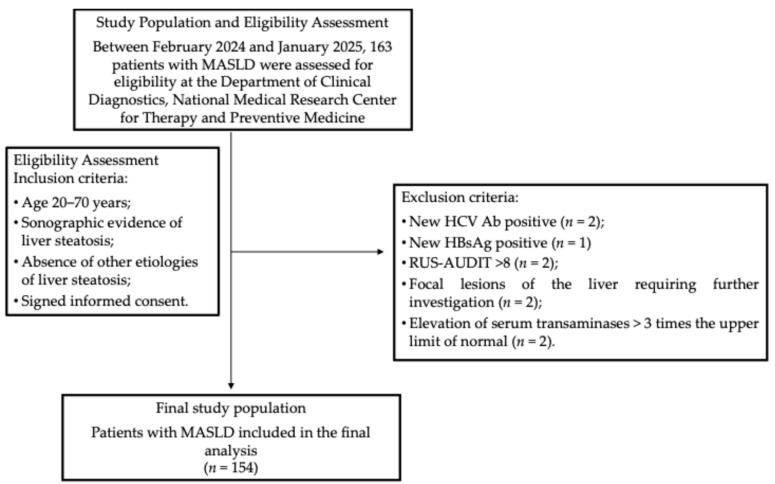
The participant flowchart. Abbreviations: HBsAg, hepatitis B surface antigen; HCV Ab, hepatitis C virus antibodies; HTN, hypertension; MASLD, metabolic dysfunction-associated steatotic liver disease; RUS-AUDIT, Russian Alcohol Use Disorders Identification Test.

During the enrollment period, 163 patients were screened for eligibility according to predefined inclusion and exclusion criteria. Nine patients were excluded for the following reasons: one tested positive for hepatitis B surface antigen (HBsAg), and two were positive for hepatitis C antibodies (HCV Ab); two had RUS-AUDIT scores ≥ 8; two demonstrated focal liver lesions on abdominal ultrasound requiring further assessment; and two exhibited transaminase levels more than three times the upper limit of normal (ULN) (Figure 1). Altogether, 154 patients were eligible for the final analysis. Their main characteristics are provided in Table 1. The median age was 56 years (47–63.5 years). Obesity was observed in ninety-nine patients (64.3%), whereas four patients (2.6%; three females and one male) had BMI values within the normal range but exhibited WC measurements exceeding 80 cm for females and 94 cm for males, indicating an increased cardiometabolic risk.

Among the 154 patients with MASLD included in this study, 140 had HTN: grade 1 HTN in 47 patients (30.5%), grade 2 HTN in 37 patients (24.0%), and grade 3 HTN in 54 patients (35.1%). T2D was diagnosed in 16 patients (10.4%), while impaired fasting glycemia (IFG) was observed in 28 patients (18.2%). Sex and age distributions did not differ significantly between HTN grades or between patients with and without IFG. However, patients with T2D were significantly older compared with non-diabetic individuals (*p* = 0.012). Laboratory test results for MASLD patients are summarized in Table 2.

The median FLI was 84.9 (IQR 61.5–94.1). Advanced liver fibrosis, defined as liver stiffness > 7 kPa, was identified in twenty-two patients (14.3%; thirteen males and nine females; *p* = 0.042).

Results of body composition analysis by DXA are provided in Table 3. Reduced appendicular skeletal muscle mass-to-body-weight ratio (ASMM/W) values were identified in 25 female patients (27.5% of the total female cohort, *n* = 91; median age 62 years [56–66]) and 16 male patients (26.7% of the total male cohort, *n* = 60; median age 52.5 years [45.3–58]).

The median FAS score was 21 (17–25). Within the MASLD cohort, 56 patients (36.4%) had FAS scores ≥ 22, indicative of clinically significant fatigue (43 females and 13 males). Clinically significant fatigue was more prevalent among female patients (*p* = 0.002). However, no significant differences were observed between patients with and without clinically significant fatigue regarding age, HTN grade, or the presence of T2D or IFG.

### 3.2. Associations Between Clinically Significant Fatigue and Clinical, Laboratory, and Anthropometric Parameters in Patients with MASLD

Statistically significant correlations between the FAS score and clinical, laboratory, and anthropometric parameters are presented in Table 4. It is worth noting that although these associations were statistically significant, the correlation coefficients were modest, suggesting that their clinical implications should be confirmed in future studies.

**Figure 2:**
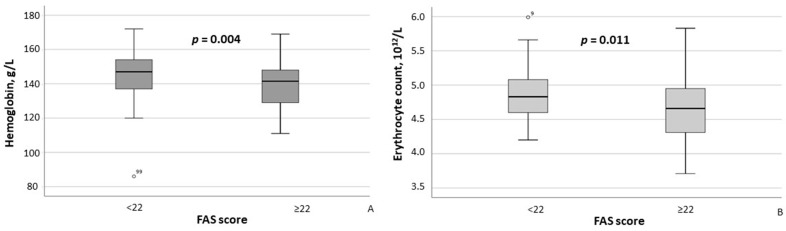
Hemoglobin concentrations (**A**) and erythrocyte counts (**B**) in MASLD patients stratified by FAS scores (≥22 vs. <22). Note: In the box plots, the central line represents the median, and the box height corresponds to IQR. Whiskers indicate the minimum and maximum values, with outliers displayed as circles. Between-group comparisons were analyzed using the Mann–Whitney U test. Abbreviations: FAS, Fatigue Assessment Scale.

**Figure 3:**
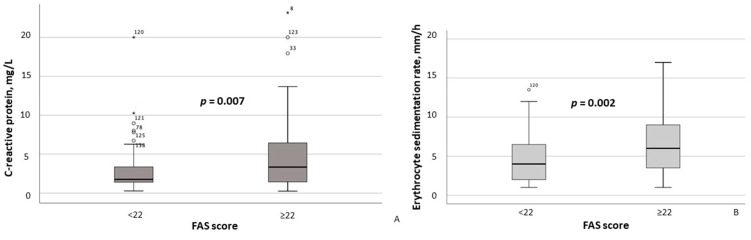
Serum C-reactive protein (**A**) and erythrocyte sedimentation rate (**B**) in MASLD patients stratified by FAS scores (≥22 vs. <22). Note: In the box plots, the central line represents the median, and the box height corresponds to the IQR. Whiskers indicate the minimum and maximum values, with outliers displayed as circles and asterisks. Between-group comparisons were analyzed using the Mann–Whitney U test. Abbreviations: FAS, Fatigue Assessment Scale.

According to the results of the between-group comparison, patients with FAS scores ≥ 22 exhibited significantly lower hemoglobin concentrations (*p* = 0.004) and erythrocyte counts (*p* = 0.011) (Figure 2A,B), as well as significantly higher CRP (*p* = 0.007) and ESR (*p* = 0.002) levels (Figure 3A,B). With respect to body composition parameters, only percent body fat differed significantly between MASLD patients with clinically significant fatigue and those without (*p* = 0.014).

**Figure 4:**
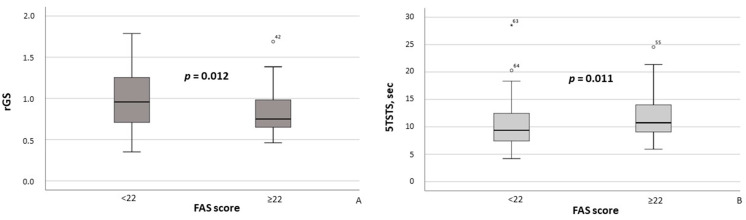
Relative grip strength (**A**) and time required to complete the Five Times Sit-to-Stand Test (**B**) in MASLD patients stratified by FAS scores (≥22 vs. <22). Note: In the box plots, the central line represents the median, and the box height corresponds to the IQR. Whiskers indicate the minimum and maximum values, with outliers displayed as circles and asterisks. Between-group comparisons were analyzed using the Mann–Whitney U test. Abbreviations: FAS, Fatigue Assessment Scale; 5TSTS, Five Times Sit-to-Stand Test; rGS, relative grip strength.

Additionally, MASLD patients with FAS scores ≥ 22 had significantly lower rGS values (*p* = 0.012; Figure 4A) and required significantly more time to complete the 5TSTS (*p* = 0.011; Figure 4B). Moreover, the presence of clinically significant fatigue was associated with higher maximum systolic blood pressure (SBP) (*p* = 0.028) and maximum diastolic blood pressure (DBP) (*p* = 0.019).

Based on stepwise logistic regression analysis adjusted for age, sex, BMI, HTN, and T2D, erythrocyte sedimentation rate emerged as the sole independent predictor of clinically significant fatigue (defined as FAS score ≥ 22) among MASLD patients in this study (odds ratio [OR], 95% confidence interval [CI]: 1.097, 1.035–1.163; *p* = 0.002). No independent associations with fatigue were observed for age, sex, BMI, HTN, or T2D.

## 4. Discussion

Fatigue is the most commonly reported symptom among patients with chronic liver diseases, including MASLD [9,10]. The reported prevalence of fatigue in MASLD patients ranges from 51.1% [3] to 67.8% [5]. This symptom frequently co-occurs with sleep disorders and depression, which collectively contribute to a significant reduction in quality of life among MASLD patients [12]. Furthermore, fatigue, daytime sleepiness, and depression may impede weight loss efforts and the achievement of therapeutic goals in MASLD. Therefore, the active identification of systemic symptoms, including fatigue, in MASLD patients is critically important, as targeted interventions can alleviate symptom severity [12] and improve quality of life and clinical outcomes. Notably, Younossi et al. [29] demonstrated that fatigue in MASLD patients was associated with a 2.3-fold increased risk of mortality over an 8-year follow-up period.

The pathogenesis of fatigue in MASLD is complex and remains incompletely understood. Several pathogenic mechanisms may contribute to fatigue in chronic liver diseases (CLDs), including MASLD, such as alterations in gut microbiota [30], immune dysregulation [31], autonomic dysfunction [7], skeletal muscle impairment [32], and disruptions in multiple neurotransmitter systems [33]. Conversely, the severity of MASLD or liver injury parameters—including the degree of steatosis, inflammation, or fibrosis—appear unrelated to the presence or severity of fatigue in MASLD patients [6,34]. Nevertheless, a recent study by A.M. Mostafa et al. [4] demonstrated a strong association between the prevalence of fatigue in MASLD patients and the prevalence and degree of liver steatosis and fibrosis as evaluated by FibroScan^®^ (*p* < 0.0001). Such discrepancies in the reported findings across studies may be explained by the use of different methodologies to assess MASLD activity. Specifically, in the study by A.M. Mostafa et al. [4], disease severity was determined using laboratory testing, including non-invasive laboratory markers and FibroScan^®^, whereas in the study by J.L. Newton et al. [6], laboratory tests and liver biopsy were employed.

Considering the aforementioned data regarding potential associations between fatigue in MASLD and systemic manifestations—particularly neurocognitive symptoms such as daytime sleepiness—as well as the proposed pathophysiological mechanisms, we conducted a study to analyze the relationship between clinically significant fatigue and laboratory parameters reflecting systemic inflammation, liver function, body composition, and muscle strength in patients with MASLD.

In this study, specific sleep assessments were not performed in enrolled MASLD patients because they are costly and time-consuming and require specialized equipment and trained staff. However, there is a high prevalence of sleep disturbances among MASLD patients with fatigue—62.5% in the MASLD group versus 19.5% in controls (*p* < 0.001), as reported by A.M. Mostafa et al. [5]—and evidence that daytime sleepiness in MASLD often results from obstructive sleep apnea characterized by intermittent hypoxic episodes. To explore a possible indirect signal of hypoxia, we compared hemoglobin and erythrocyte levels in MASLD patients with and without clinically significant fatigue. Although all values remained within normal reference ranges, patients with fatigue exhibited significantly lower levels than those without fatigue (Table 2). While these findings may be consistent with a potential contribution of hypoxia, this interpretation remains speculative, as no direct measurements of oxygenation or sleep parameters were performed. Further studies with objective assessment of sleep disorders and hypoxic burden are warranted to clarify this possible link.

Regarding muscle status, our results demonstrated a significant association between clinically significant fatigue and reduced muscle strength, whereas skeletal muscle mass assessed by DXA (ASMM/W index) did not differ between groups. This pattern aligns with recent EWGSOP2 recommendations emphasizing muscle strength as the primary diagnostic criterion for sarcopenia [23]. According to the recent work of A.J. Cruz-Jentoft et al. [35], it is important to distinguish between “sarcopenia” (i.e., the combination of reduced muscle function and mass) and “low muscle mass” alone. In our study, the observed association between fatigue and reduced muscle strength (i.e., impaired muscle function) may indicate that functional capacity plays a key role in MASLD-related fatigue. Impaired muscle function may result from neuroendocrine dysfunction, physical inactivity, and inadequate nutritional intake—including deficiencies in essential macro and micronutrients [35]—all of which have been previously documented in MASLD patients [36,37,38].

Taken together, these observations reinforce the importance of lifestyle interventions aimed not only at improving liver health but also at supporting functional capacity and reducing systemic symptoms. Given the well-documented low readiness of MASLD patients to engage in lifestyle change [39], addressing fatigue, sleep disturbances, and muscle weakness through targeted interventions may help break the vicious cycle of reduced activity and poor adherence to treatment. Nevertheless, the cross-sectional design of our study limits causal inference regarding these associations. Future longitudinal research is needed to validate our findings, clarify underlying mechanisms, and guide the development of effective interventions for MASLD-related fatigue.

Consistent with the above data, we did not observe a significant association between fatigue severity and surrogate markers of MASLD severity, including the Fatty Liver Index (FLI), liver transaminase levels, or liver stiffness measurements. However, the associations identified between clinically significant fatigue and markers of chronic inflammation, total body fat percentage, and elevated blood pressure may be interpreted within the broader context of chronic systemic inflammation [26,40,41,42] and metabolic dysregulation [42]. Moreover, higher SBP and DBP values in MASLD patients with clinically significant fatigue suggest that comorbid chronic non-communicable diseases, such as HTN and T2D, may contribute to both the occurrence and severity of fatigue. Therefore, adequate management of these comorbidities may also help alleviate fatigue in this patient population. It is worth noting that although the above-mentioned associations were statistically significant, the correlation coefficients were modest, suggesting that their clinical implications should be confirmed in future studies.

From a clinical perspective, our findings highlight the potential utility of targeted exercise programs focused on improving muscle strength, as functional impairment rather than structural muscle loss appears to play a more prominent role in fatigue. In addition, optimal management of comorbid HTN and T2D, as well as systematic screening for sleep disturbances such as obstructive sleep apnea, may represent practical strategies to reduce symptom burden in MASLD patients. Implementing these approaches in routine care could not only improve quality of life but also enhance adherence to lifestyle modification and treatment efficacy.

## 5. Study Limitations

This study has several limitations, the primary one being the small sample size and limited representation of patients with different HTN grades and T2D in our cohort, all of which may restrict the generalizability of the findings. However, the included cohort was representative of MASLD patients without significant fibrosis who received a comprehensive assessment, which enhances the applicability of the observed patterns to the broader MASLD population. Another limitation is the lack of investigation into the presence and types of sleep disorders among the MASLD patients studied. Such assessments require specialized equipment and trained personnel and are both costly and time-consuming. Nonetheless, we acknowledge that sleep disturbances may contribute to hypoxia in MASLD patients experiencing clinically significant fatigue. Conducting targeted sleep assessments in this patient population would help validate these findings and further elucidate the etiopathogenesis of fatigue in chronic liver disease. An additional limitation is the lack of histological confirmation, as liver biopsy was not performed for the diagnosis of MASLD or for evaluating disease activity and fibrosis. In accordance with EASL recommendations, non-invasive methods were used, namely standardized liver ultrasound and elastography [43]. Finally, although our findings reveal associations between fatigue and MASLD-related systemic factors, the cross-sectional design of this study limits causal interpretation. Moreover, given that multiple comparisons were conducted without statistical adjustment, the possibility of type I errors should be acknowledged, and the observed associations should be interpreted with caution. Future longitudinal research is required to clarify the directionality and underlying mechanisms of the described relationships.

## 6. Conclusions

Clinically significant fatigue is a major concern in chronic liver diseases, including MASLD. It is frequently accompanied by other systemic manifestations, such as depression and daytime sleepiness, and has a detrimental impact on quality of life and adherence to lifestyle recommendations, including dietary modification and physical activity. This, in turn, may limit the effectiveness of treatment and negatively influence prognosis.

Key findings of this study include (1) the association between fatigue and impaired muscle strength—but not reduced muscle mass—highlighting the relevance of functional capacity, which may be affected by nutritional status, physical inactivity, and neuroendocrine disturbances; (2) the observation of lower hemoglobin and red blood cell counts in patients with fatigue, which may indirectly suggest a contribution of hypoxia, although this interpretation remains speculative given the absence of direct assessments of sleep-disordered breathing or oxygenation; and (3) the link between fatigue and comorbidities such as hypertension, supporting the potential role of cardiometabolic factors in symptom severity.

Taken together, these findings emphasize the multifactorial nature of fatigue in MASLD and support the need for a comprehensive management strategy. Potential clinical applications may include targeted exercise programs to preserve muscle strength, optimal management of comorbid HTN and T2D, and screening for sleep disturbances. Further large-scale, multicenter studies are warranted to confirm these associations and inform future intervention strategies.

## Figures and Tables

**Table 1:** Main characteristics of patients with MASLD.

Parameter	Patients with MASLD (*n* = 154)
Female gender, *n* (%)	91 (59.1)
Age, years	56 (47–63.5)
BMI, kg/m^2^	32.4 (29.2–35.9)
Normal weight (BMI 18.5–24.9 kg/m^2^), *n* (%)	4 (2.6)
Overweight (BMI 25.0–29.9 kg/m^2^), *n* (%)	48 (31.2)
Obesity (BMI ≥ 30 kg/m^2^), *n* (%)	99 (64.3)
Waist circumference (all subjects), cm	107 (100–113)
Waist circumference (male), cm	111 (106–116.75)
Normal Range	≤94 cm
Patients Outside the Normal Range, *n* (%)	59 (98.3)
Waist circumference (female), cm	103.5 (97–111)
Normal Range	≤80 cm
Patients Outside the Normal Range, *n* (%)	91 (100)

Note: Continuous variables are reported as medians with IQRs, and categorical variables as counts and percentages (*n*, %). Abbreviations: BMI, body mass index; MASLD, metabolic dysfunction-associated steatotic liver disease.

**Table 2:** Laboratory findings in patients with MASLD.

Parameter	Patients with MASLD (*n* = 154)	Normal Range	Number (%) of Patients Outside Normal Range
Erythrocytes, 10^12^/L	4.77 (4.50–4.98)	—	—
Erythrocytes, 10^12^/L (male)	4.9 (4.8–5.2)	4.0–5.9	0 (0)
Erythrocytes, 10^12^/L (female)	4.6 (4.3–4.8)	3.8–5.2	0 (0)
Hemoglobin, g/L	144 (132.3–151.8)	—	—
Hemoglobin, g/L (male)	152 (147–157)	140–175	0 (0)
Hemoglobin, g/L (female)	137 (128.5–144)	123–153	0 (0)
ESR, mm/h	9 (4.25–15.75)	<20	0 (0)
Platelets, 10^9^/L	254.0 (219–296.75)	150–450	0 (0)
Leukocytes, 10^9^/L	6.4 (5.3–7.6)	5–10	0 (0)
ALT, IU/L	23.5 (16.0–36.0)	0–34	40 (26.0)
AST, IU/L	22.0 (18.0–27.0)	0–34	14 (9.1)
GGT, IU/L	31.0 (20.0–45.0)	0–38	36 (24.4)
Albumin, g/dL	4.5 (4.3–4.6)	3.5–5.2	0 (0)
Total bilirubin, µmol/L	11.6 (9.0–14.4)	3.4–20.5	6 (3.9)
Uric acid, µmol/L	351.1 (291.6–409.4)	154.7–356.9	58 (37.7)
CRP, mg/L	2.2 (1.4–4.98)	0–5	37 (24.0)
Total cholesterol, mmol/L	5.8 (4.9–6.5)	0–5	108 (70.1)
Triglycerides, mmol/L	1.5 (1.1–2.2)	<1.7	64 (41.6)
Creatinine, µmol/L	75.0 (66.2–88.3)	51–98	12 (7.8)
Glucose, mmol/L	5.7 (5.4–6.0)	3.9–5.5	94 (61.0)
HOMA-IR > 2.7, *n* (%)	83 (53.9)	0.5–1.4	—
pSWE, kPa	5.5 (4.1–6.7)	<7	22 (14.3)

Note: Continuous variables are reported as medians with IQRs, and categorical variables as counts and percentages (*n*, %). Abbreviations: ALT, alanine aminotransferase; AST, aspartate aminotransferase; CRP, C-reactive protein; ESR, erythrocyte sedimentation rate; GGT, gamma-glutamyl transferase; HOMA-IR, Homeostasis Model Assessment of Insulin Resistance; MASLD, metabolic dysfunction-associated steatotic liver disease; pSWE, point shear-wave elastography.

**Table 3:** Results of body composition analysis by dual-energy X-ray absorptiometry.

Parameter	Value
SMM, kg	44.9 (39.3–59.4)
SMM (male), kg	59.5 (52.5–61.9)
SMM (female), kg	41.4 (37.6–45.7)
ASMM, kg	23.4 (19.6–30.6)
ASMM (male), kg	30.8 (27.6–32.4)
ASMM (female), kg	20.4 (18.9–23.4)
ASMM/W (male), %	29.4 (28.1–31.0)
ASMM/W (female), %	24.3 (23.3–26.4)
Fat mass, kg	36.9 (31.9–42.7)
Fat mass (male), kg	34.4 (29.3–41.2)
Fat mass (female), kg	38.7 (33.4–44.4)
Percentage of body fat, %	42.7 (36.3–46.6)
Percentage of body fat (male), %	35.6 (31.6–37.1)
Percentage of body fat (female), %	45.6 (43.4–48.6)
Visceral adipose tissue mass, kg	1.8 (1.3–2.5)
Visceral adipose tissue mass (male), kg	2.3 (1.8–3.2)
Visceral adipose tissue mass (female), kg	1.7 (1.2–2.1)
Subcutaneous adipose tissue mass, kg	2.1 (1.6–2.7)
Subcutaneous adipose tissue mass (male), kg	1.7 (1.5–2.3)
Subcutaneous adipose tissue mass (female), kg	2.4 (1.8–2.9)

Note: Values are presented as median (interquartile range [IQR]). Abbreviations: ASMI, appendicular skeletal muscle mass index; ASMM, appendicular skeletal muscle mass; ASMM/W, appendicular skeletal muscle mass-to-body-weight ratio.

**Table 4:** Statistically significant correlations between FAS score, clinical, laboratory, and anthropometric parameters of MASLD patients.

Parameter	Positive Correlations with FAS Score		Parameter	Negative Correlations with FAS Score	
	Spearman’s Rank Correlation Coefficient, *ρ*	*p*-Value		Spearman’s Rank Correlation Coefficient, *ρ*	*p*-Value
ESR	0.277	0.001	RBC	−0.257	0.003
CRP	0.220	0.013	HGB	−0.258	0.003
Percentage of body fat	0.191	0.036	rGS	−0.187	0.032
BMI	0.181	0.036	ASMM/W	−0.193	0.033
5TSTT	0.269	0.002			
SBP	0.181	0.039			
DBP	0.199	0.025			

Abbreviations: 5TSTT, Five Times Sit-to-Stand Test; ASMM/W, appendicular skeletal muscle mass-to-body-weight ratio; BMI, body mass index; CRP, C-reactive protein; DBP, diastolic blood pressure; ESR, erythrocyte sedimentation rate; HGB, hemoglobin; RBC, red blood cells; rGS, relative grip strength; SBP, systolic blood pressure.

## Data Availability

The data presented in this study are available upon reasonable request from the corresponding authors. The data are not publicly available due to privacy restrictions.
